# Understanding Zenker’s Diverticulum Treatment: Transoral CO2 Laser Microsurgery

**DOI:** 10.7759/cureus.53329

**Published:** 2024-01-31

**Authors:** Patrícia S Sousa, Helena Silveira, Gil Coutinho, Cecília Pereira, Carla P Moura

**Affiliations:** 1 Department of Otorhinolaryngology, Centro Hospitalar Universitário São João, Porto, PRT; 2 Department of Surgery and Physiology, Faculty of Medicine, University of Porto, Porto, PRT; 3 Department of Otolarhinoryngology, Centro Hospitalar Universitário São João, Porto, PRT; 4 Department of Otorhinolaryngology, Unidade Local de Saúde do Alto Minho, Viana do Castelo, PRT; 5 Department of Otorhinolaryngology, Centro Hospitalar Universitário de São João, Porto, PRT; 6 Department of Medical Genetics, Centro Hospitalar Universitário de São João, Porto, PRT; 7 Genetics, Institute for Research and Innovation in Health/Instituto de Investigação e Inovação em Saúde, University of Porto, Porto, PRT

**Keywords:** dysphagia, co2 laser, endoscopic surgery, transoral microsurgery, zenker’s diverticulum

## Abstract

The authors present a case of a 36-year-old woman with a recurrent throat foreign body sensation and persistent dysphagia. On physical examination, a polypoid mass was visible at the postcricoid region, mobile with swallowing. A barium swallowing test confirmed the diagnosis of Zenker’s diverticulum. The patient underwent transoral CO_2 _laser microsurgery for excision of the pharyngeal pouch. At the four-month evaluation, the patient was remarkably asymptomatic and without evidence of recurrence. This clinical case illustrates in detail the endoscopic view of the pre and postoperative aspects of the pharyngeal pouch, showing a step-by-step transoral CO_2_ laser microsurgery technique, with video.

## Introduction

Zenker’s diverticulum is a dehiscence of mucosal and submucosal layers between the inferior pharyngeal constrictor muscle, superiorly, and the cricopharyngeal muscle, inferiorly, through the Killian’s triangle. High intraluminal pressure results in herniation at this level of muscular weakness triangle [1]. Dysfunction of the cricopharyngeal muscle and loss of tissue elasticity in the hypopharyngeal segment plays an important etiological role [2]. Symptoms can include prolonged dysphagia, Globus sensation, halitosis, regurgitation, and aspiration in severe cases [1,3] and its diagnosis is confirmed with a barium swallow test [4].

Surgery remains the mainstay treatment for symptomatic pouches. Open and endoscopic approaches have been used to manage this condition and a minimally invasive approach is the treatment of choice. Several endoscopic techniques are available and transoral CO_2_ laser microsurgery, introduced in 1983 [5], has been used for its efficacy, safety, and low morbidity. This clinical case shows a step-by-step endoscopic excision of a pharyngeal pouch with a CO_2_ laser, achieving good functional and anatomical outcomes.

Part of this article was previously presented as a poster at the 70^th^ Congress of the Portuguese Society of Otorhinolaryngology and Head and Neck Surgery on May 12, 2023 in Algarve, Portugal.

## Case presentation

**Figure 1:**
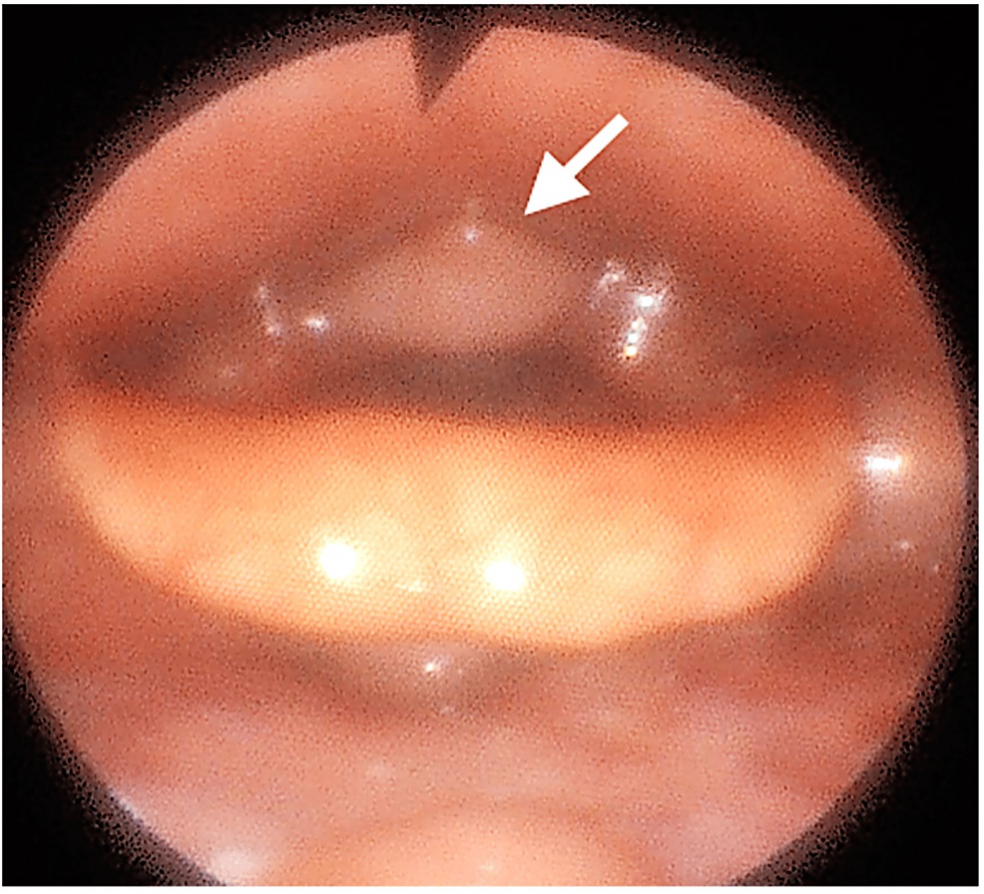
Flexible laryngoscopic examination showing a polypoid mass at the postcricoid region (arrow).

A 36-year-old woman presented to our department with a recurrent throat foreign body sensation and persistent dysphagia. Her past medical history was unremarkable. On physical examination, by flexible laryngoscopy, a polypoid mass was visible at the postcricoid region, mobile with swallowing (Figure 1, Video 1).

**Video 1:** Flexible laryngoscopic examination showing a polypoid mass at the postcricoid region, mobile with swallowing.

**Figure 2:**
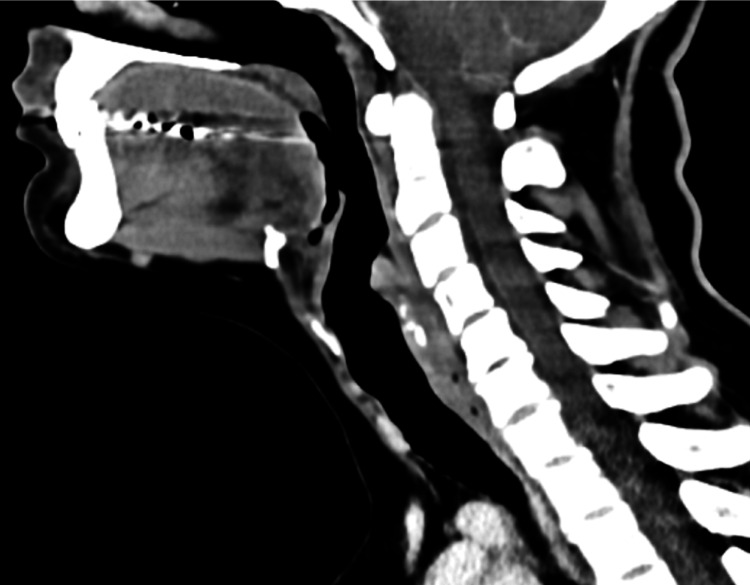
Cervical computed tomography with no clear evidence of pharyngeal pouch.

Cervical computed tomography was inconclusive (Figure 2).

**Figure 3:**
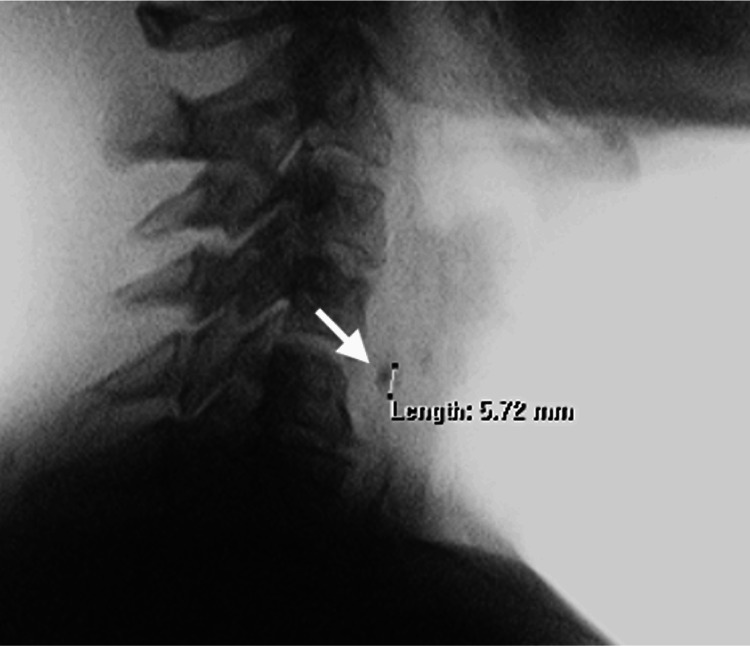
Barium swallowing test showing a small contrast retention suggestive of a pharyngeal pouch.

Barium swallowing test showed a small contrast retention of approximately 6 mm posteriorly to the plane of the cricopharyngeal muscle suggestive of Zenker’s diverticulum (Figure 3).

**Figure 4:**
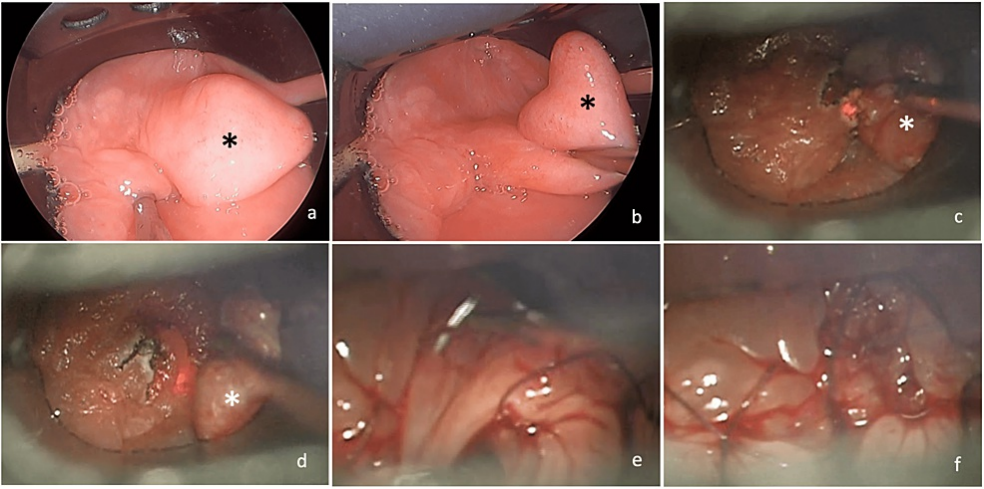
Transoral laser microsurgery (pharyngeal pouch identified with asterisk). (a, b) Pharyngeal pouch; (c, d) laser excision of the pharyngeal pouch; (e) stitches with resorbable material; (f) result after surgery.

The patient underwent transoral CO_2_ laser microsurgery under general anesthesia for excision of the pharyngeal pouch. The procedure was performed with a rigid laryngoscope (diverticuloscope) under microscopic observation. The first step was to identify the diverticulum, the postcricoid region, and the esophageal lumen (Figures 4a, 4b). Diverticulum excision was performed with a CO_2_ laser at 5 watts: it was excised by its pedicle through the mucosal and submucosal layers, with identification of the muscular layer and division of its fibers (Figures 4c, 4d), keeping in mind not to transect the buccopharyngeal fascia. Hemostasis was performed with CO_2_ laser and suction cautery. Stitches with resorbable material (vicryl 5-0) were made to approximate the redundant mucosa (Figures 4e, 4f). Complete step-by-step surgery can be watched in Video 2.

**Video 2:** Transoral laser microsurgery with excision of pharyngeal pouch.

**Figure 5:**
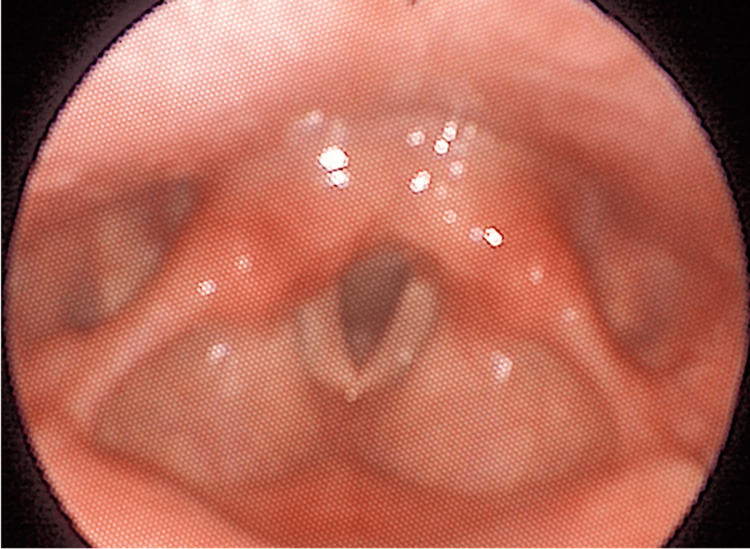
Four-month evaluation with flexible laryngoscopic examination without evidence of recurrence.

The surgical procedure took approximately two hours. Minimum edema was visible, intravenous steroids were administrated at the immediate postoperative period, and no further steroid medication was administrated. After the procedure, the patient was on intravenous antibiotics (amoxicillin and clavulanate acid) and kept in a nasogastric tube for 48 hours. On the first two days after surgery, the patient had mild pharyngeal discomfort/pain. On the third day, an indirect laryngoscopy was performed, and a small area of fibrin was visible (surgical site) with no inflammatory or infectious signs the patient was started on a liquid/soft diet with good tolerance, and the nasogastric tube was removed. No videofluoroscopy was performed as the patient tolerated the diet very well and there was no evidence of complications. The discharge occurred four days after surgery without complications, with a recommendation to maintain a soft diet for two weeks. At the four-month evaluation, the patient was remarkably asymptomatic and without evidence of recurrence (Figure 5).

## Discussion

The treatment of Zenker’s diverticulum should be tailored to each patient, and several techniques are shown to be effective throughout the literature. For small and asymptomatic diverticula, watchful waiting can be an option [4]. For symptomatic pouches surgery is the mainstay treatment. Surgical approaches include endoscopic and open transcervical [3]. The open transcervical approach, although highly effective with up to 90% symptomatic relief [1] and a low recurrence rate (4.2% vs. 18.4%) [6], has more morbidity and complications than the endoscopic approach [1,6,7]. The endoscopic approach is the treatment of choice in patients in which the procedure can be managed by a minimally invasive approach, depending on the anatomic characteristics of the diverticulum and the patient’s comorbidities [6]. Furthermore, the CO_2_ laser technique is particularly useful for small diverticula, variable within the literature, ranging from 2.5 to 4.3 cm [2-4]; however, it is not exclusive. For large diverticula, it can be difficult to perform endoscopic surgery, so it is very important to consider not only the size of the diverticulum but also the anatomic characteristics of the patient, like exposure to a rigid endoscope. Several endoscopic techniques are available, such as laser, diathermy, or stapling, and transoral laser microsurgery (CO_2_, KTP, or thulium laser) has been widely used for its efficacy, safety, and low morbidity.

The endoscopic technique principle is to perform a diverticulotomy by separating the common wall between the esophagus and the pouch [7] and the laser technique allows to perform a cricopharyngeal myotomy [1,8], with a midline vertical incision [4,8]. In this case, it was possible to perform a diverticulectomy endoscopically because of the small size and well-defined pedicle of the pharyngeal pouch. The CO_2_ laser provides great cutting power and minimizes thermal damage to adjacent structures, promoting a safer and faster healing [7]. Laser microsurgery is shown to be more effective [1,7], to have better symptomatic improvement for dysphagia and regurgitation [1], and to have a better quality of life than endoscopic stapling [2]. Some authors report no difference regarding recurrence [2], while others suggest 10% more recurrence rate for endoscopic stapling [1].

**Figure 6:**
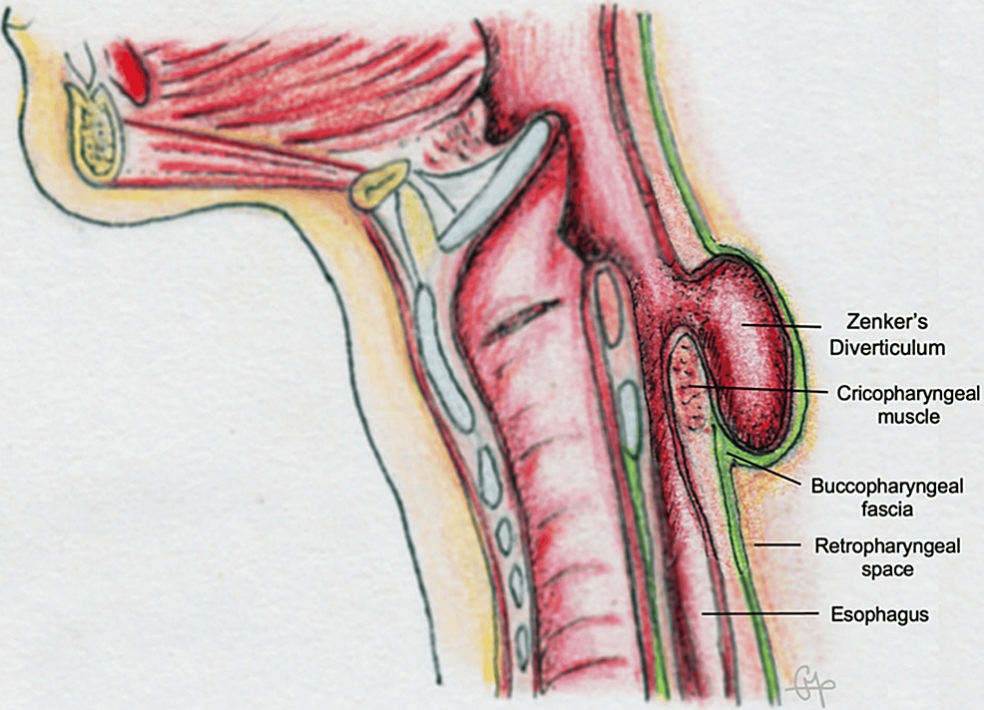
Anatomic scheme of Zenker’s diverticulum and its relations with the buccopharyngeal fascia. Courtesy: Dr. Cecília Pereira, M.D.

Regardless of the surgical approach, it is crucial to bear in mind that the diverticulum is contained within the buccopharyngeal fascia (Figure 6), essential to separate the upper aerodigestive tract from the retropharyngeal space [7,8]. Great care must be taken to preserve this fascia in order to prevent mediastinitis [4,8].

## Conclusions

In the endoscopic era, transoral CO_2_ laser microsurgery can be a safe option, highly effective, and with low morbidity for the treatment of Zenker’s diverticulum. This report illustrates in detail the anatomy of the pharyngeal pouch, showing a step-by-step surgical technique, with the preservation of the buccopharyngeal fascia, allowing easier understanding and safety in performing the surgical procedure, with good functional and anatomical postoperative outcomes.
